# Record linkage under suboptimal conditions for data-intensive evaluation of primary care in Rio de Janeiro, Brazil

**DOI:** 10.1186/s12911-021-01550-6

**Published:** 2021-06-15

**Authors:** Claudia Medina Coeli, Valeria Saraceni, Paulo Mota Medeiros, Helena Pereira da Silva Santos, Luis Carlos Torres Guillen, Luís Guilherme Santos Buteri Alves, Thomas Hone, Christopher Millett, Anete Trajman, Betina Durovni

**Affiliations:** 1 Instituto de Estudos em Saúde Coletiva, Universidade Federal do Rio de Janeiro, Avenida Horácio Macedo, s/n Ilha do Fundão – Cidade Universitária, Rio de Janeiro, RJ CEP 21941-598 Brasil; 2 Secretaria Municipal de Saúde do Rio de Janeiro, Rio de Janeiro, Brazil; 3 Public Health Policy Evaluation Unit, Imperial College London, London, UK; 4 Department of Preventive Medicine, School of Medicine, University of São Paulo, São Paulo, 01246-903 Brazil; 5 Center of Data and Knowledge Integration for Health (CIDACS), Instituto Gonçalo Muniz, Fundação Oswaldo Cruz, Salvador, Brazil; 6 Programa de Pós-Graduação em Clínica Médica e Mestrado Profissional em Atenção Primária à Saúde, Federal University of Rio de Janeiro, Rio de Janeiro, Brazil; 7 TB International Centre, McGill University, Quebec, Canada; 8 Centro de Estudos Estratégicos, Fundação Oswaldo Cruz, Rio de Janeiro, Brazil

**Keywords:** Medical record linkage, Data accuracy, Brazil, Primary healthcare

## Abstract

**Background:**

Linking Brazilian databases demands the development of algorithms and processes to deal with various challenges including the large size of the databases, the low number and poor quality of personal identifiers available to be compared (national security number not mandatory), and some characteristics of Brazilian names that make the linkage process prone to errors. This study aims to describe and evaluate the quality of the processes used to create an individual-linked database for data-intensive research on the impacts on health indicators of the expansion of primary care in Rio de Janeiro City, Brazil.

**Methods:**

We created an individual-level dataset linking social benefits recipients, primary health care, hospital admission and mortality data. The databases were pre-processed, and we adopted a multiple approach strategy combining deterministic and probabilistic record linkage techniques, and an extensive clerical review of the potential matches. Relying on manual review as the gold standard, we estimated the false match (false-positive) proportion of each approach (deterministic, probabilistic, clerical review) and the missed match proportion (false-negative) of the clerical review approach. To assess the sensitivity (recall) to identifying social benefits recipients’ deaths, we used their vital status registered on the primary care database as the gold standard.

**Results:**

In all linkage processes, the deterministic approach identified most of the matches. However, the proportion of matches identified in each approach varied. The false match proportion was around 1% or less in almost all approaches. The missed match proportion in the clerical review approach of all linkage processes were under 3%. We estimated a recall of 93.6% (95% CI 92.8–94.3) for the linkage between social benefits recipients and mortality data.

**Conclusion:**

The adoption of a linkage strategy combining pre-processing routines, deterministic, and probabilistic strategies, as well as an extensive clerical review approach minimized linkage errors in the context of suboptimal data quality.

## Background

A variety of administrative data is available for analysis in Brazil, including live births, mortality, outpatient clinics, and publicly funded hospital care. Health information is produced at the various administration levels (federal, state, municipal) using the same systems and under the same standards, yielding National Databases [1].

Record linkage has been used in specific projects conducted by the Ministry of Health, State and Municipal Health Secretariats, as well as university researchers. Databases with personal identifiers are assigned to the latter, after approval by a research ethics committee. To access the databases, the researchers must meet many requirements aimed to ensure privacy and data security [2].

Linking Brazilian databases demands the development of algorithms and processes to deal with various challenges including the large size of the databases as well as the low number and poor quality of personal identifiers available to be compared [3, 4]. In addition, some characteristics of Brazilian names also make the linkage process prone to errors. Homonyms are usual, despite the high frequency of double given names and multiple family names. Family names may include the full extension or only parts of either the father and mother’s family names, making it difficult to identify members of the same family [5].

Despite the increased popularity of record linkage in Brazil, only few initiatives linked various health databases [2, 6, 7] to undertake data-intensive health research [8]. To carry out data-intensive research about the impacts on health indicators of the expansion of primary care in Rio de Janeiro City, we created an individual-level dataset linking social benefits recipient, primary health care, hospital, and mortality data.

A reform of the public health system in Rio de Janeiro City started in 2009. By then, the coverage of primary health care (PHC) was 3.5%, reaching 55% in 2015 [9]. The reform was based on the National Policy of Primary Care of the Ministry of Health, known as Family Health Strategy (FHS). FHS comprised new forms of funding and both administrative and conceptual changes, led by the government of Rio de Janeiro City. Family Health teams deployed in defined catchment areas to deliver care to a fixed population should cover the essential attributes defined by Starfield [10], giving attention to the patient’s point of contact with the health system, delivering comprehensive care and follow-up and coordinating care needed outside the primary care. The strategic municipal plan was designed to achieve PHC coverage of around 40% at 2013 and 70% by 2016. Communities and social control were included in the planning to strengthen the partnership that would result in better health outcomes for those in greater need. The population covered by the FHS reached more than 3.8 million at the end of 2016, with 1116 teams.

This study aims to describe and evaluate the quality of processes used to create an individual-linked database for data-intensive research on the impacts on health indicators of the expansion of primary care in Rio de Janeiro, Brazil.

## Methods

### Data sources

Table 1 displays an overview of the data sources used, which are also briefly described below.

**Table 1:** Overview of data sources

Database	Social Benefits National Registry (Cadastro Único—CadU)	Family Health Registry (Sistema de Cadastro da Estratégia de Saúde da Família—FHR)	Eletronic Medical Registry (Prontuário Eletrônico de Pacientes—EMR)	National Hospital Admission System (Sistema de Informações Hospitalares—SIH)	National Mortality Information System (Sistema de Informações sobre Mortalidade—SIM)^a^
Time period	2008–2014	2009–2016	2011–2017	2011–2016	1999–2016
Database size	1,680,700 registrations—1,679,320 individuals	3,732,688 registrations—3,594,623 individuals	17,764,475 consultations—16,808,685 single consultations	1,787,601 hospitalizations	2,263,964 deaths
Personal identifiers	Name; Date of birth; Mother’s name; Address; Social Security Number (Cadastro de pessoa física—CPF); National register for social benefit (Número de inscrição social—NIS)	Name; Date of birth; Mother’s name; Address; Social Security Number (Cadastro de pessoa física—CPF); National register for social benefit (Número de inscrição social—NIS)	Name; Date of birth; Social Security Number (Cadastro de pessoa física—CPF)	Name; Date of birth; Mother’s name; Address	Name; Date of birth; Mother’s name; Address
Content variables	Demographic characteristics, detailed socioeconomic, and housing data	Demographic characteristics, detailed socioeconomic, housing data, self-reported chronic diseases, a summary of health care used, vital status	Patient data (e.g., demographic characteristics), the reason for encounter, laboratory and test requests, treatment plans	Patient data (e.g., demographic characteristics); hospital data (e.g., public or private); hospitalization data (e.g., admission and discharge dates, intermediary unit use, diagnosis upon discharge)	Demographic characteristics, socioeconomic data, date of death, cause of death, data on mother in the case of fetal death, and death of children under 1 year

^a^Database from Rio de Janeiro State; all others from Rio de Janeiro City

#### The Social Benefits National Registry (Cadastro Único para Programas Sociais do Governo Federal—CadU)

The Social Benefits National Registry (Cadastro Único para Programas Sociais do Governo Federal—CadU) is the database where people who want to receive welfare and social benefits from the Brazilian government are registered. These benefits include the cash-transfer program (Programa Bolsa Família—PBF), the low-cost energy social program (Tarifa Social de Energia Elétrica—TSEE), and a continuous pension benefit for the elderly and handicapped (Benefício de Prestação Continuada—BPC) [11, 12]. The registry is composed of both individual and household data including schooling and education, employment and income, and the household characteristics. The social security number (Cadastro de Pessoa Física—CPF) is an individual’s unique identifier that can be used for direct linkage to other databases containing the same identifier. We obtained an extraction of the CadU dataset for 2015 which included all individuals registered up to the 31st Dec 2014. This database was the origin of the study population, which we linked to the other databases. Before linking CadU to other databases, we created an identifier to allow the unique identification of 1380 individuals (0.08% of the records; Table 1) who changed households (e.g., due to marriage) and presented duplicated records (see data pre-processing).

#### The Family Health Registry (FHR) and the Electronic Medical Registry (EMR)

The Electronic Medical Registry (EMR) was implemented to be the main clinical, administrative and epidemiological data management tool of primary care. It was designed to allow integration with the Brazilian Primary Care Information System (SIAB—Sistema de Informação da Atenção Básica), nowadays replaced by the e-SUS [13, 14]. The Family Health Registry (FHR) is part of the SIAB. It is composed of personal, socioeconomic, housing data, and a summary of health care use of individuals living and/or being followed by each family health team. The EMR was designed to be used by physicians, nurses and community health workers. Health indicators, both epidemiological and pay-for-performance ones, were obtained directly from the EMR. Personal data included the CPF (the unique identifier also present in the CadU) which permits a deterministic linkage between the EMR and the FHR databases. However, one person could be registered in two or more different health units, after moving from one territory to another. We linked the CadU database to FHR/EMR datasets to evaluate primary care exposure.

#### The Hospital Admissions Information System (Sistema de Informação Hospitalar—SIH)

The Hospital Admissions Information System (SIH) is an administrative database for the authorization of hospital admissions, including payments and auditing in the public health system [15], which only covers the hospitalizations publicly funded, therefore limiting its use to the general population. The causes of hospital admissions are coded according to the 10th revision of the International Statistical Classification of Diseases and Related Health Problems (ICD-10), thus making the SIH a valuable source of morbidity data. The SIH was used to identify the number and causes of hospital admissions from 2011 to 2016 of individuals registered in CadU.

#### The Brazilian Mortalilty Information System (Sistema de Informação sobre Mortalidade—SIM)

The Brazilian Mortalilty Information System (SIM) started in 1975 in order to unify the various Death Declaration Forms being used in the states. The SIM database records individual deaths certificates, including description of the causes of death and the population profile [15]. The coding of the causes is based on the ICD-10. The SIM database was used to identify who died before 2011 (exclusion criteria) and deaths and causes of death from 2011 and 2016 of individuals registered in CadU.

### Data pre-processing

We used PostgreSQL [16] and OpenReclink [17] to pre-process the databases. The databases were available in Xbase, TXT, and CSV formats. First, we standardized the attribute separators, as they changed over time in some databases. Then, we imported each database into PostgreSQL and ran various routines to clean the names and address attributes. We removed punctuation marks, special characters, leading and trailing white space characters, stop words, and invalid terms. We replaced multiple white space characters by one white space character, converted all letters into upper case, and Unicode characters into ASCII characters. We standardized date formats and coding schemes of the matching attributes. Finally, we created a deterministic linkage key concatenating the soundex phonetic code of the first individual’s given name, the soundex phonetic code of the individual’s second segment of the name (second double given name or first family name), the soundex phonetic code of the individual’s last family name, the sex and the date of birth. We also created two new unique identifiers for the identification, respectively, of each record and each individual. The latter was necessary whenever there were multiple records associated with the same individual. After running the deterministic routines (see below), we exported the databases to OpenReclink and carried out the parsing of the names and date of birth attributes.

### Record linkage

We linked the CadU database to the FHR, SIH, and SIM databases, one at a time. In all of these processes, we combined deterministic and probabilistic linkage, plus clerical review approaches. We also linked FHR to EMR records, however, performing only the deterministic procedure. A pilot study showed a minimal gain as well as a high cost in terms of the number of candidate record pairs that needed to be manually reviewed when we added the other approaches. The reasons for this low performance are the absence of the mother’s name attribute in EMR data, and the greater efficiency of the deterministic approach, since the common personal identifiers of FHR and EMR datasets are generated by the same computerized system using a shared table. The electronic medical records were stored in ten different files, each containing data from health facilities located in the same region of the city. Due to the large size of these files, we linked each of them to the FHR database separately.

**Fig. 1:**
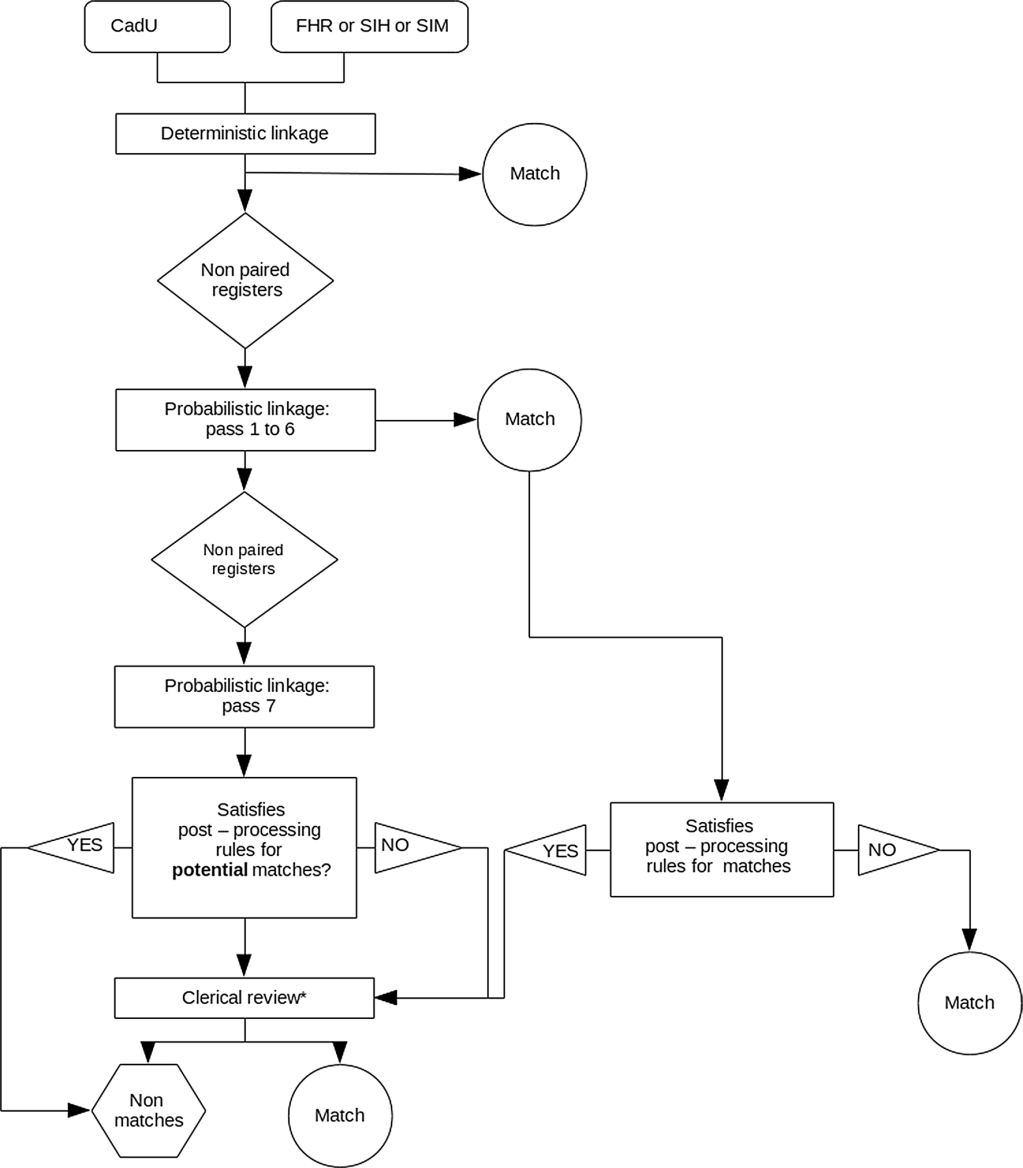
Flow diagram of the record linkage process

We adopted a sequential strategy, sending to the probabilistic approach only the records for which a match was not identified in the deterministic phase (Fig. 1). Likewise, we only sent to a subsequent probabilistic pass the records for which a match was not found in a previous pass. The only exception was the linkage between the CadU and the SIH databases. Because various hospitalizations registered in the SIH database might refer to a single individual recorded in CadU (one-to-many situation), we keep all records of the CadU database throughout the whole record linkage process.

#### Deterministic record linkage

We carried out the deterministic linkage using PostgreSQL [16]. The rules used to classify record pairs as matches varied according to the attributes available in the databases to be linked (Table 2). Our team developed the rules empirically based on the experience acquired in the clerical review of previous projects.

**Table 2:** Rules applied in the deterministic approach to classifying pairs as matches

Rules	
(1) Exact agreement on the deterministic linkage key	
(2) Exact agreement on the social security number (CPF)	
(3) Exact agreement on the National register for social benefit (NIS)	
(4) Exact agreement on date of birth	
(5) The Levenshtein distance of the individual’s name < 3	
(6) The Levenshtein distance of the mother’s name < 3	
(7) Exact agreement on the individual’s name	
Linkage processes’ criteria	
CadU versus FHR	(1, 5 and 6) OR (2, 5 and 6) OR (3, 5 and 6) OR (2 and 4) OR (3 and 4)
CadU versus SIH	(1, 5 and 6)
CadU versus SIM	(1, 5 and 6)
FHR versus EMR	(1 and 5) OR (2 and 5) OR (4 and 7)

The Levenshtein edit distance measures the minimum number of edits (insertions, deletions, or substitutions) required to change one name string into the other [18]

#### Probabilistic record linkage

We used OpenReclink [17] for the probabilistic linkage. We applied a seven-pass blocking strategy using indexing keys formed by different combinations of the following attributes: soundex phonetic code of the individual’s first name, soundex phonetic code of the individual’s last name, year of birth and sex. In the first blocking pass, we used an indexing key formed by the concatenation of the four attributes. From the second to the forth blocking passes, the indexing key was formed, concatenating three attributes at a time. We evaluated the similarity of the candidate record pairs generated in the first five blocking steps by comparing the individual’s name, the mother’s name, and the individual’s date of birth. Since Brazilians frequently have multiple family names, and it is usual to record the last name and to present only the initials of the others, we carried out a sixth blocking pass. Using the same indexing key applied in the first blocking pass, we compared the candidate record pairs generated using the individual’s first given name, the individual’s last family name, the sex, and the date of birth. Finally, we carried out a seventh blocking pass using the same indexing key applied in the first blocking pass but comparing the candidate record pairs generated using the individual’s name and date of birth (Table 3).

**Table 3:** Description of the probabilistic linkage blocking passes

Blocking pass	Indexing key	Comparison	Calculated score range according to estimated weights	Score cutoff values		
				CadU × HR	CadU × IH	CadU × IM
1	Soundex first name + Soundex last name + sex + birthyear	Individual name + mother’s name + birth date	− 38.71 to 34.99	28.60	33.97	21.90
2	Soundex first name + sex + birthyear	Individual name + mother’s name + birth date	− 38.71 to 34.99	31.67	32.88	31.91
3	Soundex last name + sex + birthyear	Individual name + mother’s name + birth date	− 38.71 to 34.99	34.00	34.55	34.11
4	Soundex first name + soundex last name + sex	Individual name + mother’s name + birth date	− 38.71 to 34.99	32.73	33.00	31.70
5	Soundex first name + soundex last name + birthyear	Individual name + mother’s name + birth date	− 38.71 to 34.99	32.21	33.12	34.44
6	Soundex first name + soundex last name + sex + birthyear	First individual name + last individual name + mother’s name + birth date	− 53.51 to 45.38	41.30	43.86	44.23
7	Soundex first name + soundex last name + sex + birthyear	Individual name + birth date	− 32.57 to 21.9	17.20	17.68	17.32

Social Benefits National Registry (Cadastro Único—CadU); Family Health Registry (Sistema de Cadastro da Estratégia de Saúde da Família—FHR); National Hospital Admission System (Sistema de Informações Hospitalares—SIH); National Mortality Information System (Sistema de Informações sobre Mortalidade—SIM)

We used the Levenshtein edit distance to compare names, which measures the minimum number of edits (insertions, deletions, or substitutions) required to change one name string into the other [18], and an exact character-by-character algorithm to compare the date of birth. Each candidate pair of records had a composite weight assigned, calculated as the sum of the agreement or the disagreement weights for each field being compared. We estimated the linkage weights through the Expectation–Maximization (EM) algorithm [19] and defined a composite weight upper threshold empirically in each blocking pass (Table 3). The candidate record pairs generated in the six first blocking steps that presented a composite weight equal to or higher than the upper threshold were classified as matches. In the seventh blocking pass, they were classified as potential matches (Fig. 1).

#### Probabilistic record linkage post-processing

We post-processed all the record pairs classified as matches (first to sixth blocking pass) or potential matches (seventh blocking pass) using PostgreSQL [16]. All record pairs classified as matches were reclassified as potential matches and sent to clerical review if: (a) the individual’s name length was less than or equal to 20; or (b) the soundex phonetic code of the second segment of the individual’s name disagreed. We also reclassified as non-matches the candidates record pairs classified as potential matches if: (a) the individual’s first given name was common (frequency of the soundex phonetic code > 5); and (b) the individual’s name length was less than or equal to 20; and (c) the Levenshtein edit distance of the mother’s name was greater than or equal to 10; and (d) the Levenshtein edit distance of the address was greater than or equal to 12. These criteria identified record pairs that were unlike to be true matches, avoiding sending then to be manually reviewed (Fig. 1).

#### Clerical review (manual review)

**Fig. 2:**
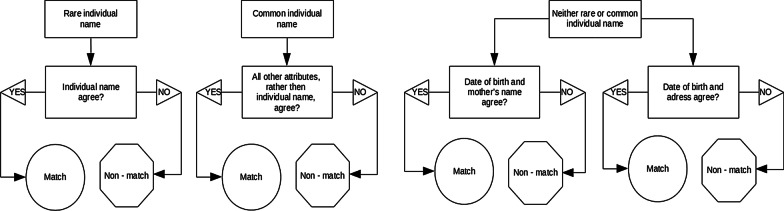
General rules for record pairs classification

Eight reviewers manually assessed the candidate record pairs classified as potential matches. Each reviewer was assigned a batch of non-overlapping candidate pairs. The reviewers were trained and evaluated by one research expert in clerical review, who was also responsible for their supervision. They assessed the same attributes used in the probabilistic process, along with the address. We let the reviewers decide each attribute's agreement, and the final resolution of the candidate record pair (match or non-match) without using any set of detailed criteria. We only oriented the use of few general rules for record pairs classification, which were developed empirically based on the experience gained in previous projects, as follows: (a) if the individual’s name is rare, then the record pair should be classified as a true match, even in the presence of disagreements in one or more other attributes; (b) if the individual’s name is common, then the record pair should be classified as a true match only if all other attributes agreed; (c) the individual’s name is not common neither rare, the record pair should be classified as a true match if the date of birth and either the mother’s name or the address agreed (Fig. 2). The name is considered common if formed by a given name and only one surname, and the name or the surname is frequent in Brazil [20, 21]. On the other hand, it should be considered rare if: (a) formed by a given name and two surnames and none of them are frequent in Brazil [20, 21]; or (b) present three or more surnames. Doubts were discussed with the supervisor, who was responsible for resolving the record pair status (match or non-match). Every week, the supervisor discussed with all the reviewers the dubious situations so as to establish guidelines for future decisions.

The training consisted of a 3-h session when the rules were presented along with real examples. After that, the reviewer had to classify correctly at least 90% of 200 pairs of records to be approved.

### Preparation of datasets for analysis

The final phase was the merge of the matched record pairs generated in each approach and the identification and elimination of record pairs wrongly assigned as matches. We manually reviewed any duplicated record pairs of the same individual in a one-to-one match situation (eg., CadU vs. SIM). Likewise, we sent to manual review five or more repeated records of the same individual in the CadU versus SIH linkage process (one-to-many case). Finally, we removed all personal identifiers, keeping only the new unique identifiers created in the pre-processing phase.

### Linkage quality evaluation

We rely on manual review as the gold standard to evaluate the linkage quality. Two reviewers who did not participate in the initial clerical review process evaluated the samples of records pairs. The reviewers were aware of the status of the record pair assigned in the different approaches (deterministic, probabilistic, and clerical review), and they could either agree or disagree. In the case of disagreement, the supervisor decided the final status (match or non-match). Hence, pairs of records automatically classified as matches in the deterministic or the probabilistic approaches were manually reviewed for the first time. In contrast, the pairs of records classified as match or non-match in the clerical review approach were reviewed a second time by a different reviewer.

We drew from each approach, without replacement, simple random samples from record pairs classified as matches (*N* = 744). We used this sample size to estimate the odds ratios for potential factors associated with linkage errors that we intend to evaluate in a future analysis. Likewise, we drew a simple random sample from record pairs classified as non-matches in the clerical review approach.

We estimated the false match proportion (records from different individuals that are linked) of each approach (deterministic, probabilistic, clerical review) and the missed match proportion (records from the same individual that are not linked) of the clerical review approach. For the linkage between the CadU and the SIM databases, we determined, in addition, the recall proportion using as the gold-standard the information about the vital status registered on the FHR. First, we selected all record pairs from the linkage between CadU and FHR with a date of death between 1999 and 2016 (N = 4179). In doing that, in the CadU database, we were able to add the information about each individual's vital status registered in the FHR database. Then, we evaluated how many of the individuals identified as deceased in the FHR (the gold standard) were also identified as deceased through the linkage between CadU and SIM. We calculated the recall proportion for the entire population and according to using the FHS services (yes/no). It was the only situation where we combined information from three databases (CadU, SIM, and FHR).

Each member of a family registered with FHS teams, at least in theory, should be recorded in the FHR database. Nevertheless, 297,280 individuals recorded in the CadU database, who were in a family with a FHS registered individual, did not have a match record in the FHR database. This find could be due to missing data in the FHR database or linkage error. To clarify this question, we drew a sample of 744 records from these CadU records and extensively manually searched them in the FHR database.

### Ethical approval

Approval for this study was obtained from the Brazilian National Commission for Ethics in Research (Comissão Nacional de Ética em Pesquisa [CONEP])—number 2.689.528.

## Results

Table 4 shows the completeness of the personal identifiers in each data source. Sex, date of birth, and the individual’s name had no or very little missing data in all data sources, except for the individual's name in hospitalization database (SIH), which also presented the highest proportion of missing data in the mother's name attribute. The address was missing in around 20% of the records in the CadU and EMR databases and in about 10% of the mortality database (SIM). Half of the records in the CadU database had the social security number filled, while the primary identifier of that database (NIS) was missing in more than 90% of the FHR and EMR records.

**Table 4:** Completeness of personal identifiers available for record linkage

Database	Social Benefits National Registry (Cadastro Único—CadU)	Family Health Registry (Sistema de Cadastro da Estratégia de Saúde da Família—FHR)	Eletronic Medical Registry (Prontuário Eletrônico de Pacientes—EMR)	National Hospital Admission System (Sistema de Informações Hospitalares—SIH)	National Mortality Information System (Sistema de Informações sobre Mortalidade—SIM)^a^
	N = 1,680,700	N = 3,732,688	N = 17,764,475	N = 1,787,601	N = 2,263,964
Identifiers	%	%	%	%	%
Name	100	100	99.2	96.6	99.8
Mother’s name	99.6	100	(–)	90.2	96.6
Date of birth	100	100	100	100	97.2
Sex	100	100	100	100	100
Address	82.3	98.8	80.0	98.6	88.6
Social security number	56.5	82.7	84.0	(–)	(–)
NIS	99.6	7.3	7.3	(–)	(–)

Social security number (Cadastro de Pessoa Física—CPF)

National register for social benefit (Número de Inscrição Social—NIS)

(–) Attribute not available in the database

^a^Excluding fetal deaths and deaths of children under 1-year-old

**Fig. 3:**
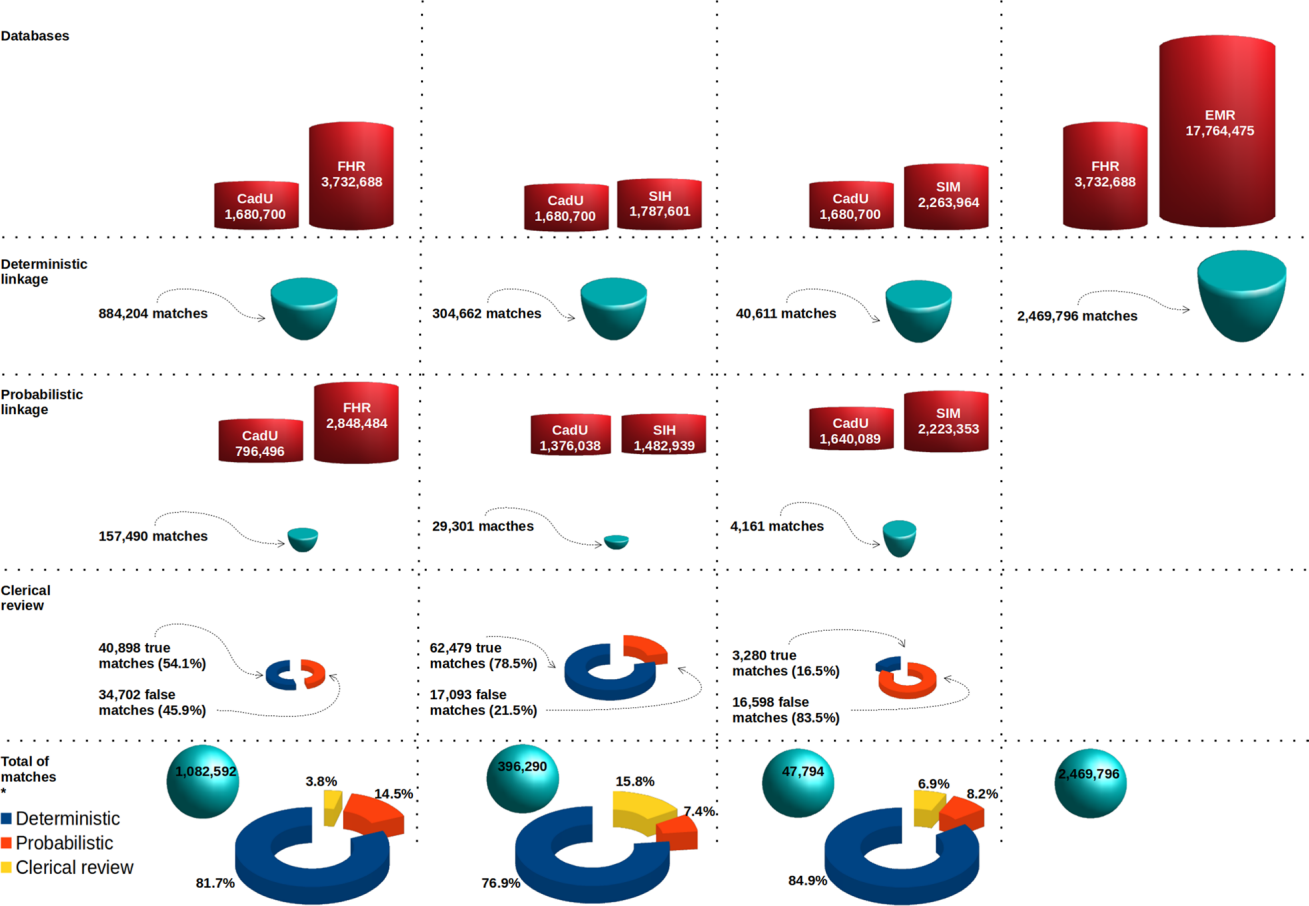
Overview of matches identified in each approach. “Total of matches” excludes record pairs identified as wrongly assigned as matches in the procedure of preparing datasets for analysis

In all linkage processes, the deterministic approach identified most of the matches. The linkage of the CadU database to the FHR database identified the highest proportion of matches deterministically. In contrast, the linkage of the CadU database to the SIH database presented the lowest percentage of matches detected through the deterministic approach and the highest percentage identified through clerical review (Fig. 3). That linkage generated the largest volume of pairs to be reviewed and had the highest proportion of pairs classified as matches (78.6%) in this approach. We observed an opposite pattern for the linkage between the CadU and the SIM databases, which presented the lowest volume of revised pairs and the lowest proportion of pairs classified as matches (16.9%). Approximately half the pairs from the linkage of the CadU to the FHR databases sent to review were correct matches (Fig. 3).

We estimated the false match and the missed match proportions using the manual review as the gold standard. The false match proportion was around 1% or less in almost all approaches except for the clerical review in the linkage between the CadU and SIH databases (3.89%) and the CadU and SIM databases (2.55%) (Table 5). The missed match proportions in the clerical review approach of all linkage processes were also low, as follows: CadU versus FHR 2.96% (95% CI 1.86–4.44); CadU versus SIH 1.21% (95% CI 0.55–2.28); CadU versus SIM 0.27% (95% CI 0.03–0.96).

**Table 5:** False match proportion acoording to the linkage approach

Measures	CadU vs. FHR % (95% CI)	CadU vs. SIH % (95% CI)	CadU vs. SIM % (95% CI)	FHR vs. EMR % (95% CI)
Deterministic (N = 744)	1.07 (0.47–2.11)	0	0.13 (0.00–0.07)	0.67 (0.22–1.56)
Probabilistic (N = 744)	0.27 (0.03–0.97)	0	0.67(0.22–1.56)	(–)
Clerical review (N = 744)	0.40 (0.08–1.17)	3.89 (2.63–5.55)	2.55 (1.54–3.95)	(–)

(–) Linkage approach did not execute

False match (records from different individuals that are linked), missed match (records from the same individual that are not linked)

We estimated a recall of 92.5% (95% CI 91.7–93.3; [3864/4179]) for the linkage between the CadU and SIM databases, based on the information about the vital status registered on the FHR. The recall proportion did not vary significantly according to the use of the FHS services: yes = 92.8% (95% CI 91.8–93.7; [2857/3080]); no = 91.6% (95% CI 90–93.3; [1007/1099]).

Finally, analyzing a sample of the 297,280 individuals recorded in the CadU database, who were in a family with an ESF registered individual and did not have a match record in the FHR database, we found that 89.7% of them (N = 667/744) were missing in the FHR database.

## Discussion

Even under suboptimal conditions, we managed to create an individual-linked database for data-intensive research with low linkage error rates by adopting a linkage strategy that combined multiple approaches. Our strategy is in line with a recent guideline for linking data for health service, prepared for the Agency for Healthcare Research and Quality (AHRQ) [22]. It recommends the combined use of deterministic and probabilistic approaches in contexts of poor data quality to improve record linkage accuracy more efficiently.

We created the individual-linked database to evaluate the impacts on health indicators of the expansion of primary care in Rio de Janeiro, Brazil. We used CadU as the study population and linked it to FHR/EMR datasets to evaluate primary care exposure. To evaluate hospitalizations and mortality, we linked CadU to SIH and SIM databases, respectively.

We used the same general strategy to link the CadU database to the FHS, hospitalization (SIH), and mortality data (SIM). However, the proportion of matches identified in each approach varied. The linkage between CadU and FHR databases found more than 90% of the matches through the deterministic approach, while the linkage between CadU and SIH databases identified three quarters. We implemented the deterministic approach using simple rules aiming to minimize false match errors [23]. The rules included the exact agreement on one personal identifier combined with distance metrics of names. Since the CadU and FHR databases have more personal identifiers in common, we were able to apply different rules in the deterministic routine. On the other hand, the SIH database presented the lower completeness of the individual's and the mother's names attributes, which probably impaired the accuracy of the deterministic routine. The deterministic approach is particularly prone to missed match errors when an exact agreement is used, and there is a lack of personal identifiers that are complete and accurate [24].

The number and quality of personal identifiers also influence the probabilistic approach [24], which may explain the fact that the linkage of the CadU to the SIH databases presented the lowest proportion of matches being found through the probabilistic approach. The probabilistic approach had the highest proportion of matches identified in the CadU and SIM databases’ linkage. In our study, the completeness of the individual’s and the mother’s name was higher in SIM than in SIH. SIM is the oldest Brazilian Information System. Over the years, it has improved its completeness and consistency [15, 25].

The clerical review is the most labor-intensive and time consuming process in record linkage [26]. When high-quality matching variables are available, it is possible to achieve excellent discrimination of matches and non-matches using deterministic, probabilistic, or a combination of the two processes without needing to carry out the manual review of the potential matches [23]. It was not the case in our study, with a significant number of matches being identified through clerical review in all linkage processes, particularly in the linkage between the CadU and SIH databases. This linkage process generated the higher number of potential matches sent to manual review, which can be explained by the large size of the SIH database, and the inability of the deterministic and probabilistic approaches in discriminating matches from non-matches [26]. Manual classification decisions can differ from reviewer to reviewer, and even for the same reviewer when asked to classify the same potential match more than once [26]. In our study, the clerical review process resulted in a low proportion of missed match and false match errors. We adopted quality assurance measures that might have contributed to this result, including training, evaluation, and supervision of the reviewers.

Pre-processing was the second approach in terms of time and resource consuming in our study. Data cleaning is considered an essential step for improving record linkage in the scenario of poor data quality [22]. However, two studies carried out in Australia [27] and in the USA [28] showed that data cleaning routines decreased the missed match error, but increased the false match error. The AHRQ’s guidelines [22] recommend that the extent of data cleaning (minimum to high) should be tailored according to the quality of data (high vs. low) and the research question (exploratory analysis vs. hypothesis test).

We tailored all the approaches to minimize false match errors. Unlike missed match errors, false match errors are positively correlated with the size of the databases to be linked [29]. Moore et al. [30] showed that false positive errors bias the incidence rate more significantly than missed match errors. Likewise, false match errors have a greater impact on the risk ratio than missed match errors, when the exposure and the outcome misclassification errors are independent, and the outcome misclassification is non-differential with regards to the exposure levels [30, 31]. Although some studies [24], but not all [4], found non-differential linkage errors, in the current analysis, the recall proportion did not vary significantly according to the use of the FHS service, suggesting a non-differential bias.

To evaluate the impacts on health indicators of the expansion of primary care, we used record linkage to classify both the exposure to primary care and the outcomes (for instance, mortality) [32]. Therefore, our analysis might be vulnerable to information bias due to dependent misclassification. However, many factors might have decreased dependence. Firstly, the number and quality of personal identifiers used varied according to the linkage process. Secondly, we estimated a different false match and missed match proportions in each approach and linkage process. Finally, as we linked the CadU to the FHR database, and the FHR to the EMR database, we were able to carry out sensitivity analyses, applying different specifications for the exposure, based on FHS registration or FHS services usage [32].

One limitation of our study was the use of the manual review as the gold standard for estimating the false match and the missed match proportions. However, to minimize errors due to the inherent subjectivity of manual classification, the supervisor decided the final status (match or non-match) whenever the reviewer of the validation sample assigned a discordant class from the initial classification. The linkage strategy adopted was complex, making it difficult to obtain a representative group of records classified as non-matches. Hence a further limitation was the lack of assessment of the recall measures for almost all linkage processes, except for the linkage between the CadU and the SIM databases. For this linkage, the gold standard was the information about the vital status registered on the FHR. Therefore, the analysis was restricted to the individuals registered in the CadU database who were found in the FHR database. However, we believe that the results observed for this particular subset of the CadU individuals may be generalized to the whole CadU population, as selection bias is unlikely. We carried out the linkage between the CadU and the SIM databases without knowing which individuals were also registered in the FHR database. Also, we estimated that about 90% of the individuals recorded in the CadU database, who were in a family with a FHS registered individual and did not have a match record in the FHR database, were missing in the FHR database. This result suggests that significant linkage errors are less likely to explain missed matches in the linkage of the CadU to the FHR databases. Finally, the reviewers of the linkage quality evaluation were aware of the record pairs status assigned in the initial review process, which might have contributed to overestimate the accuracy measures.

Newcombe [33], a pioneer in record linkage, pointed out that the art of record linkage lies in the ability to introduce automated classifier refinements based on insights gained through the complex and intuitive process of clerical review. Alternative methods based on supervised machine learning classification techniques [26] have been used in record linkage projects achieving accurate results [34]. However, one of the challenges of using such techniques is the lack of representative samples of labeled training data [26]. As a result of the extensively clerical review process carried out in our study, we generated a high-quality training dataset, which we intend to use to explore the accuracy of different machine learning classifiers in the Brazilian context of suboptimal data quality.

In conclusion, the adoption of a linkage strategy combining pre-processing routines, deterministic, and probabilistic strategies, as well as an extensive clerical review approach, minimized linkage errors in the context of suboptimal data quality. Although we reported our experience of linking Brazilian databases, we believe that the processes we developed to deal with various challenges can help Population Data Science researchers worldwide.

## Data Availability

The datasets generated during and/or analyzed during the current study are not publicly available due to the privacy of personal information essential to link the databases but are available from the corresponding author on reasonable request.

## Abbreviations

AHRQ: Agency for Healthcare Research and Quality
BPC: Benefício de Prestação Continuada
CadU: Cadastro Único (Social Benefits National Registry)
CONEP: Comissão Nacional de Ética em Pesquisa (Brazilian National Commission for Ethics in Research)
CPF: Cadastro de Pessoa Física (social security number)
EM: Expectation–maximization algorithm
EMR: Electronic Medical Registry
FHR: Family Health Registry
FHS: Family Health Strategy
ICD-10: International Statistical Classification of Diseases and Related Health Problems-10th revision.
NIS: Número de inscrição social (national register for social benefit)
PBF: Bolsa Família Program
PHC: Primary health care
SIAB: Sistema de Informação da Atenção Básica (Brazilian primary care information system)
SIH: Sistema de Informação Hospitalar (hospital admissions information system)
SIM: Sistema de Informação sobre Mortalidade (Brazilian mortalilty information system)
TSEE: Tarifa social de energia elétrica
